# Patients with unexplained physical symptoms have poorer quality of life and higher costs than other patient groups: a cross-sectional study on burden

**DOI:** 10.1186/1472-6963-13-520

**Published:** 2013-12-17

**Authors:** Lyonne NL Zonneveld, Mirjam AG Sprangers, Cornelis G Kooiman, Adriaan van ’t Spijker, Jan JV Busschbach

**Affiliations:** 1 Department of Psychiatry, Section Medical Psychology and Psychotherapy, Erasmus Medical Center, PO Box 2040, 3000, CA Rotterdam, The Netherlands; 2 Department of Anesthesiology, Academic Medical Center, University of Amsterdam, PO Box 22660, 1100, DD Amsterdam, The Netherlands; 3 Department of Medical Psychology, Academic Medical Center, University of Amsterdam, PO Box 22660, 1100, DD Amsterdam, The Netherlands; 4 Department of Psychotherapy, Riagg Rijnmond, Stationsplein 2, 3112, HJ Schiedam, The Netherlands

**Keywords:** Absenteeism, Burden, Chronic disease, Costs, Healthcare utilization, Presenteeism, Production loss, Quality of life, Somatic symptom disorder, Somatoform disorder, Unexplained physical symptoms

## Abstract

**Background:**

To determine whether healthcare resources are allocated fairly, it is helpful to have information on the quality of life (QoL) of patients with Unexplained Physical Symptoms (UPS) and on the costs associated with them, and on how these relate to corresponding data in other patient groups. As studies to date have been limited to specific patient populations with UPS, the objective of this study was to assess QoL and costs in a general sample of patients with UPS using generic measures.

**Methods:**

In a cross-sectional study, 162 patients with UPS reported on their QoL, use of healthcare resources and lost productivity in paid and unpaid work. To assess QoL, the generic SF-36 questionnaire was used, from which multidimensional quality-of-life scores and a one-dimensional score (utility) using the SF-6D scorings algorithm were derived. To assess costs, the TiC-P questionnaire was used.

**Results:**

Patients with UPS reported a poor QoL. Their QoL was mostly decreased by limitations in functioning due to physical health, and the least by limitations in functioning due to emotional problems. The median of utilities was 0.57, and the mean was 0.58 (SD = .09).

The cost for the use of healthcare services was estimated to be €3,123 (SD = €2,952) per patient per year. This cost was enlarged by work-related costs: absence from work (absenteeism), lower on-the-job productivity (presenteeism), and paid substitution of domestic tasks. The resulting mean total cost was estimated to be €6,815 per patient per year.

**Conclusions:**

These findings suggest that patients with UPS have a high burden of disease and use a considerable amount of healthcare resources. In comparison with other patient groups, the QoL values of patients with UPS were among the poorest and their costs were among the highest of all patient groups. The burden for both patients and society helps to justify the allocation of sufficient resources to effective treatment for patients with UPS.

**Trial registration:**

Nederlands Trial Register, NTR1609

## Background

Unexplained Physical Symptoms (UPS), such as chronic fatigue syndrome, are physical symptoms that cannot be explained on the basis of a known medical condition. The estimated prevalence of UPS ranges from 20 to 74% in primary care [1], and is 52% in secondary care [2,3]. As UPS reduces not only Quality of Life (QoL) of patients [4-9] but also increases costs [9-11], UPS is a burden to patients and society alike. Information on the burden of diseases is important, as the allocation of healthcare resources to patient groups is partly based on who has ‘the highest burden’ , which is considered an egalitarian and thus ‘fair’ distribution of healthcare resources [12,13]. Therefore, information is needed on the QoL of patients with UPS, on the costs associated with them, and on how these relate to corresponding data in other patient groups.

Studies [4-9] show that the Quality of Life (QoL) of patients with UPS is poor. To explore how this relate to the QoL in other patient groups, the different domains of QoL should be summarized into a one-dimensional weighted score, so-called ‘utility’ [14,15]. In a utility, one represents full health and zero is equivalent to death. We found three studies that calculated utilities for patients with UPS [4,16,17]. One study [16] was conducted in a rehabilitation clinic and defined patients with UPS as ‘psychosomatic’ patients, who were treated for depression, anxiety, and other mental disorders. They found a mean utility of 0.54. The second [4] was conducted in primary care and defined UPS as a somatoform disorder, but it excluded the most prevalent somatoform disorder: the undifferentiated somatoform disorder [7]. They found a mean utility of 0.70. The third study [17] was conducted in primary care and recruited patients – some of whom were considerably disadvantaged socioeconomically – from general practices in London. They found a mean utility of 0.47.

Costs to society are incurred by the healthcare services attended by patients with UPS. One study [9] found $4,700 annual healthcare expenditure per patient; another found $5,678 [10]. Little is known about the less visible societal costs associated with UPS due to lost productivity in paid and unpaid work. For chronic fatigue syndrome, Reynolds et al. [11] estimated that the annual societal costs for lost labor force and household productivity in the United States were $9.1 billion, which amounted to approximately $20,000 per patient [11]. It is unknown whether the cost associated with chronic fatigue syndrome is representative for the overall group of patients with UPS.

Since studies to date have been limited to specific patient populations with UPS, the objective of this study was to assess QoL and costs in a general sample of patients with UPS using generic measures. We investigated the objective with the following research questions:

1. What is the QoL of patients with UPS?

2. What are the healthcare-related costs associated with patients with UPS?

3. What are the work-related costs associated with patients with UPS?

4. What is the mean total cost per patient with UPS per year?

## Methods

### Study design

The cross-sectional study was part of a randomized controlled trial on the effectiveness of cognitive-behavioral group training for patients with UPS [18]. As part of the baseline measurement, patients were asked to complete self-report questionnaires on QoL and costs. A more detailed description of the study protocol [19] has been published elsewhere.

### Study population

Between February 2005 and September 2008, patients were recruited in general practices, outpatient clinics at general hospitals, and at the Riagg Rijnmond, a secondary community mental-health service for the greater Rotterdam area (the Netherlands). Both the attention of physicians and of patients with UPS was drawn to the randomized controlled trial on the effectiveness of cognitive-behavioral group training for UPS. Physicians’ attention was drawn to the group training by periodical postcards informing them when and how they could refer patients to the group training. Patients’ attention was drawn to the group training by announcements in local newspapers and on websites of patients’ associations, in which they were asked to make an appointment with their physician to discuss referral when interested. General practitioners and medical specialists were asked to refer those patients, who were aged between 18 and 65 years, and whose physical symptoms, according to their clinical judgment, could not sufficiently be explained on the basis of a known medical condition. Patients were included if they signed the informed consent and if their UPS fulfilled the DSM-IV criteria for an undifferentiated somatoform disorder or a chronic pain disorder.

We chose to use undifferentiated somatoform disorder and chronic pain disorders for the definition of UPS and excluded the other somatoform disorders. The reason to define UPS in this way was, that the prevalence of undifferentiated somatoform disorder and chronic pain disorder is higher [7] and their DSM-IV criteria have more similarity than the other somatoform disorders. By defining UPS in this way, we had the largest, most homogeneous, and clinical relevant sample based on the internationally agreed DSM-IV criteria developed for research, clinical practice, and education. Our definition for UPS seems to be supported by the new edition of DSM, as the DSM-5 also combined some but not all somatoform disorders and labeled them complex somatic symptom disorder.

To verify whether UPS fulfilled the DSM-criteria for undifferentiated somatoform disorder or chronic pain disorder, we used the Structured Clinical Interview for DSM-IV Axis I Disorders (SCID-I) [20], a semi-structured validated interview for making the major DSM-IV Axis I diagnoses. Patients were excluded if they did not provide informed consent, or if poor language skills or handicaps such as cognitive impairment prevented them from accomplishing the tasks required by the study.

### Outcome measures

The QoL in different domains was measured using the 36-item Medical Outcomes Study Short-Form General Health Survey (SF-36) [21], a validated and reliable self-report questionnaire for QoL, designed to be used across a wide range of different populations [22]. The responses to the 36 items of this questionnaire are converted into eight multi-item subscales: ‘Physical functioning’ , ‘Role functioning physical’ , ‘Bodily pain’ , ‘General health’ , ‘Vitality’ , ‘Social functioning’ , ‘Role functioning emotional’ , and ‘Mental health’. Raw scale scores are linearly transformed into a 0-to-100 scale, a higher score indicating a better QoL.

To extract utilities from the SF-36, a smaller version of the SF-36 has been developed by using eleven SF-36 items to cover six dimensions (‘SF-6D’): ‘Physical functioning’ , ‘Role limitations’ , ‘Social functioning’ , ‘Pain’ , ‘Mental health’ , and ‘Vitality’ [23]. Each dimension has between four and six response levels, thereby providing 18,000 possible quality-of-life states. A selection of these states was valued as more or less preferable by a representative sample of the UK general population using a valuation technique known as ‘Standard Gamble’. On the basis of their valuations, the utilities for all 18,000 possible health states have been estimated using regression models. As a result, the SF-6D outcomes can now be transformed into a utility where one represents full health, and zero is equivalent to death.

Costs were measured using the 2002 version of the Trimbos/iMTA Questionnaire for Costs associated with Psychiatric Illness (TiC-P), a self-report questionnaire for assessing the costs of illness: it has 29 questions and semi-fixed-response alternatives [24].

The first part of the TiC-P measures the healthcare-related costs incurred through the use of healthcare services and medication over the past four weeks. Hereby patients are asked whether they had a consult with a particular healthcare provider. If they had, the next question inquires after the number of consultation with this healthcare provider during the past four weeks. By multiplying the number of consultations with the matching reference prices for the particular consultation described in the manual for costing studies [25] and the TiC-P manual [26], the healthcare-related costs are estimated.

The second part, which is based on the short form of the Health and Labor Questionnaire, measures the work-related costs caused over the past two weeks by absenteeism (absence from work), by presenteeism (reduced efficiency at work), and by substitution of domestic tasks.

•For the work-related cost due to absenteeism, patients are asked whether they have a paid job. If they have, the next questions inquire after the number of working hours and working days per week. Subsequently, patients are asked whether they had any disability days due to their health problems during the past two weeks, and, if so, the next questions inquire after the number of disability days in this period and the date when reporting ill. By multiplying the number of lost working hours due to absence through illness with the average productivity cost per patient based on gender and age described in the TiC-P manual [26], the work-related cost due to absenteeism is estimated.

•For the work-related cost due to presenteeism, patients with a paid job are asked whether they were less efficiently at work due to their health problem during the past two weeks. If they were, the next question inquires after the number of hours lost to this inefficiency. By multiplying the number of lost working hours due to reduced efficiency at work with the average productivity cost per patient based on gender and age, the work-related cost due to presenteeism is estimated.

•For the work-related cost due to substitution of domestic tasks, patients are asked whether they were hindered in their domestic tasks due to their health problems during the past two weeks. If they were, the next questions inquire after by whom they were replaced and how many hours were substituted. By multiplying the number of substituted hours by the matching reference price for the particular paid professional [25], the work-related cost due to substitution of domestic tasks by paid professionals is estimated.

The TiC-P manual [24] recommends to assess costs attributable to health problems in general instead of the target disease only for the following reasons: 1) comorbidity is common in psychiatric illness; and 2) patients have difficulties in distinguishing healthcare consumption and production loss due to the target disease from those due to other health problems.

### Ethics

The study has been approved by the Erasmus Medical Research Ethics Committee and has been registered in the Dutch Trial Register (NTR1609) [27]. Patients in this study gave written informed consent.

### Statistical analyses

First, the different domains of QoL were assessed by calculating the means and the standard deviations of the SF-36 subscales. The overall QoL was analyzed by calculating the median, mean and standard deviation of SF-6D.

Second, the healthcare-related costs were indexed for 2007 using the price index figures available online on Statistics Netherlands [28,29], after which the mean and the standard deviation of the sum of healthcare-related costs per year were calculated.

Third, the work-related costs of patients with UPS comprise costs caused by loss of productivity in paid work (absenteeism and presenteeism) and costs caused by loss of productivity in unpaid work (substitution of domestic tasks).

For the calculation of work-related cost due to absenteeism, the friction-cost method was used [24,30]. The friction-cost method assumes that every working person who is absent for 23 weeks and more is replaced by a formerly unemployed person and is therefore excluded from the cost calculations. The work-related cost due to absenteeism was indexed for 2007 using price index figures for labor available online on Statistics Netherlands [31], after which the mean and the standard deviation of this cost per year were calculated. The work-related cost due to presenteeism was also indexed for 2007 using price index figures for labor available online on Statistics Netherlands [31], after which the mean and the standard deviation of this cost per year were calculated. The work-related cost of productivity loss in unpaid work was calculated only if domestic tasks were substituted by paid professionals [25]. The cost for paid substitution of domestic task was indexed for 2007 using price index figures for labor available online on Statistics Netherlands [31], after which the mean and standard deviation of this cost per year were calculated.

Fourth, the mean total cost per year was the sum of annual costs due to healthcare utilization and lost productivity in paid and unpaid work.

## Results

### Patient characteristics

Table 1 shows the characteristics of the 162 patients enrolled in the study. Due to missing answers, the SF-6D could not be calculated for five patients (3%).

**Table 1:** Patients’ characteristics

**Socio-demographic characteristic**	**n**	**%**
**Gender**		
Female	131	81%
Male	31	19%
**Age in years *(mean, SD)***	*45*	*11*
**Nationality**		
Dutch	141	87%
Other	21	13%
**Marital status**		
Married or living with partner	110	68%
Unmarried, divorced or widowed	52	32%
**Highest education completed**		
Primary school or less	14	9%
Lower vocational or general secondary education	54	33%
Intermediate vocational or higher general secondary education	57	35%
Higher vocational, pre-university, or university education	36	22%
Missing	1	1%
**Employment**		
Employed	73	45%
Unemployed	89	55%
**Number of working hours per week of patients with paid work *(mean, SD)***	*23.9*	*10.9*
**Number of working days per week of patients with paid work *(mean, SD)***	*3.9*	*1.2*
**Clinical characteristic**	**n**	**%**
**Classification of UPS by SCID-I**		
Undifferentiated somatoform disorder	63	39%
Chronic pain disorder	99	61%
**Duration of UPS in years *(median, interquartile range)***	*9*	*3-16*
**Number of comorbid DSM-IV Axis I disorders**		
No comorbid DSM-IV Axis I disorder	95	59%
One or more comorbid DSM-IV Axis I disorders	67	41%
**Number of comorbid DSM-IV Axis II disorders**		
No comorbid DSM-IV Axis II disorder	113	70%
One or more comorbid DSM-IV Axis II disorders	47	29%
Missing	2	1%
**Referrer**		
Primary medical service	82	51%
Secondary medical service	51	31%
Secondary mental service	29	18%

### Quality of life

Table 2 summarizes the SF-36 subscale means and standard deviations of patients with UPS. The score on the subscale ‘Role functioning physical’ was the lowest, and the score on the subscale ‘Role functioning emotional’ the highest. This means that patients reported that their QoL was decreased the most by limitations in functioning due to physical health, and the least by limitations in functioning due to emotional problems. However, the standard deviation of the subscale ‘Role functioning emotional’ was the largest of all subscales.

**Table 2:** Patients’ means and standard deviations on SF-36 subscales

**SF-36 subscale**	**Mean**	**SD**
Physical functioning	50.9	23.80
Role functioning physical	15.6	27.30
Bodily pain	33.2	19.57
General health	37.9	17.70
Vitality	33.4	17.86
Social functioning	49.2	24.42
Role functioning emotional	65.0	42.76
Mental health	62.7	20.14

When the QoLwas summarized in utilities, the utilities ranged from 0.37 to 0.96. The median of utilities was 0.57 and the mean was 0.58 (SD = .09).

### Healthcare-related costs

As Table 3 shows, patients with UPS consulted general practitioners, medical specialists and physiotherapists most. Almost all patients took medication, the most commonly being for pain without inflammation inhibition, for pain with inflammation inhibition, and for depression/anxiety. The mean of healthcare-related costs was estimated to be €3,122.93 (SD = €2,952.25) Per Patient Per Year (PPPY).

**Table 3:** Healthcare visits and healthcare-related costs per patient with UPS per year

**Healthcare service**	**Percentage of patients using the service**	**Mean visits per patient per year**	**Mean costs per patient per year in €**^**1)**^	**Percentage of total costs**
General practitioner	58.64%	15.57	420.31	13.46%
Medical specialist	38.89%	8.43	584.96	18.73%
Physiotherapist	35.80%	14.12	490.25	15.70%
Alternative health practitioner	22.84%	5.22	248.30	7.95%
Company doctor	18.52%	2.97	80.16	2.57%
Secondary community mental-health service	11.11%	2.41	396.94	12.71%
Private psychiatric/psychotherapeutic practice	11.11%	2.49	211.08	6.76%
Social worker	9.26%	2.01	125.74	4.03%
Psychiatric outpatient clinic	8.64%	1.12	82.64	2.65%
Self-help group	1.85%	0.32	16.26	0.52%
Inpatient hospital care service	0.62%	0.24	100.98	3.23%
Substance-abuse outpatient care service	0.62%	0.08	2.13	0.07%
Day hospital care service	0.00%	0.00	0.00	0.00%
**Medication**	**Percentage of patients using medication**	**Mean number different medication per patient**	**Mean costs per patient per year in €**^**1)**^	**Percentage of total costs**
Medication	85.19%	2.81	363.17	11.63%
Medication with prescription		2.25		
Medication without prescription		0.56		
**Total costs per patient**			3122.93	100.00%

^**1)**^Costs are adjusted for base year 2007.

### Work-related costs

Of the 162 patients with UPS, 73 (45%) had paid work, 34 of whom (46.6%) reported absence from work; 9 (12.3%) were partially absent, and 25 (34.3%) fully absent. In 15 of the 73 (21%) employed patients, this full absence had passed the end of the 23-week friction period. The mean cost of productivity lost to absenteeism was estimated to be €5,334.72 (SD = €11,980.95) per employed patient per year, and €2,403.92 (SD = €8,442.89) PPPY (mean of the total study group).

Most working patients with no or only partial absence reported lower on-the-job productivity; only seven (14.6%) did not. The mean number of working hours lost per working patient due to this presenteeism was two hours per week (mean = 2.0;SD = 4.2) – over six percent (mean = 6.3;SD = 12.7) of their contracted hours. The mean cost of presenteeism was estimated to be €1,899.16 (SD = €5,248.08) per employed patient per year, and €855.79 (SD = €3,635.31) PPPY (mean of the total study group).

Disability and reduced efficiency were also reported in the performance of domestic tasks. Of the 162 patients with UPS, 89 (55%) asked other people to perform some domestic tasks for them for a duration of four hours (mean = 4.3;SD = 12.3) per week. For only 21 of these 89 patients (24%), domestic tasks were performed by paid professionals for a duration of almost three hours (mean = 2.8;SD = 1.9) per week. The mean cost of paid substitution of domestic tasks was estimated to be €433.27 (SD = €1,478.16) PPPY (mean of the total study group).

### Total cost

The mean total cost was estimated to be €6,815.91 (SD = €10,923.14) PPPY.

## Discussion

### Main findings: the burden of patients with UPS

The objective of our study was to assess QoL and costs in a general sample of patients with UPS using generic measures. Patients with UPS reported a poor QoL. Their QoL was decreased the most by limitations in functioning due to physical health, and the least by limitations in functioning due to emotional problems.

The healthcare-related cost was estimated to be €3,122.93 (SD = €2,952.25) PPPY, which was based on the number of visits to healthcare services and the medication use. While the eight medical specialist visits per year in our study were comparable with the seven visits found by Barsky et al. [10], the 15 primary care visits per year in our study were much higher than the four found by Barsky et al. [10]. Our findings on primary care visits were more comparable with studies of high-utilizing patients with UPS [8,32], in which the mean number of visits per year ranged from 13.6 [32] to 15.9 [8]. In contrast to the high number of primary care visits, the mean number of 0.24 hospital days PPPY in our study was extremely low. Smith et al. [9] found a mean of 1.9 hospital days per patient per three months. This difference might be related to the fact that our sample was drawn from a non-institutionalized population, which thus underrepresented hospitalizations.

The work-related cost due to absenteeism was estimated to be €2,403.92 (SD = €8,442.89) PPPY. The work-related cost due to absenteeism was based on the estimate of 67 disability days per employed patient per year. This number of disability days found in our study was comparable with the results of an earlier study [6], which found 18.2 disability days per three months in primary care patients with UPS.

The work-related cost due to presenteeism was estimated to be €855.79 (SD = €3,635.31) PPPY and that due to paid substitution of domestic tasks was €433.27 (SD = €1,478.16) PPPY.

The mean total cost per patient with UPS per year was estimated to be €6,815.91.

### Our main findings in relation to the literature: the burden of patients with UPS in comparison with other patient groups

As the allocation of healthcare resources to patient groups is partly based on who has ‘the highest burden’ , the burden of patients with UPS was compared with other patient groups and the general population whose QoL or costs had been measured in earlier studies using comparable outcome measures.

The SF-36 subscale means of patients with UPS were compared with those found in patients with major depression, in patients with cancer, and in the general population [22,33], resulting in Additional file 1. According to Osoba [34], the Minimally Important Difference (MID) for SF-36 subscale scores is 10. MID is defined as ‘the smallest difference in score in the domain of interest which patients perceive as beneficial and which would mandate, in the absence of troublesome side effects and excessive cost, a change in the patient’s management’ [35]. In comparison to other patient groups, patients with UPS had generally lower SF-36 subscale means, meaning a poorer QoL. However, they did not differ a MID from both other patient groups on the following subscales ‘Vitality’ , ‘Role functioning emotional’ and ‘Mental health’. On the ‘Vitality’ subscale, patients with UPS and those with major depression had comparable means that differed more than the MID from patients with cancer who reported higher vitality. On the ‘Role functioning emotional’ subscale, patients with UPS and those with cancer had comparable means that differed more than the MID from patients with major depression who reported lower emotional functioning. On the ‘Mental Health’ subscale, patients with UPS and those with cancer had comparable means that differed more than the MID from patients with major depression who reported lower mental health. Earlier studies that compared QoL of patients with UPS with that of patients with major depression [4-7] also found that patients with UPS reported a poorer QoL in the physical domain and a relatively better QoL in the mental domain than patients with major depression. Earlier studies that compared QoL of patients with UPS with that of patients with a medical diagnosis found that patients with UPS reported a poorer QoL in all domains than those with a medical diagnosis [8,9].

The SF-6D median of patients with UPS was compared with those found in patients with mental disorders and chronic physical conditions and in the general population [36-39], resulting in Additional file 2. According to Walters and Brazier [40], the MID for SF-6D scores is 0.04. In comparison with other patient groups, patients with UPS had generally a lower utility, meaning a poorer overall QoL. The utility of patients with UPS was comparable to that of patients with the following disorders: panic disorder, dysthymia, social phobia, any mood disorder, and cancer. The utility of patients with UPS was more than the MID higher than that of patients with major depression.

The healthcare-related cost of patients with UPS was compared with the findings of the Cost of Illness study in the Netherlands 2007 [41]. For this comparison, the cost had to be expressed as a percentage of total Dutch annual healthcare expenditure as shown in Additional file 3. The proportion of total Dutch annual healthcare expenditure associated with UPS was estimated to be over four percent. This healthcare expenditure was compared with those of all main disease categories and with those of patients with specific diseases and the general population [41], resulting in Additional files 4 and 5. In comparison with the healthcare expenditure of all main disease categories, the expenditure of patients with UPS was comparable to that of the category ‘blastomas; cancer and benign tumors’ (see Additional file 4). In comparison with the healthcare expenditure of patients with specific diseases and the general population, only patients with dementia and those with a mental retardation had higher healthcare expenditure than patients with UPS (see Additional file 5).

The work-related cost due to absenteeism in paid work of patients with UPS was compared with that found in earlier studies in the general population, the healthy workforce, and the workforce with chronic illness [42-45], using the mean number of disability days and the mean percentage of absenteeism per patient per year. The percentage of absenteeism is the number of lost working days due to absenteeism divided by the number of working days according to labor contract and expressed as a percentage. As Additional file 6 shows, patients with UPS had the highest number of disability days relative to that of other reference populations, even if patients who had passed the end of the friction period were excluded. Also, patients with UPS had the largest percentage of absenteeism compared with that of other reference populations.

The work-related cost due to presenteeism in paid work of patients with UPS was compared with that found in the general population and the workforce with chronic illness [43,44,46]. As Additional file 7 shows, the mean cost of patients with UPS was higher than that of patients with psychiatric disorders but lower than that of patients with rheumatic arthritis.

The work-related cost due to productivity loss in unpaid work was calculated only if the unpaid work of patients with UPS was substituted by paid professionals. Because other studies summed up the costs for both unpaid and paid substitution [44,47], the resulting cost for substitution of unpaid work of patients with UPS could not be compared with that of different reference populations.

### Limitations and strengths in the study

A number of limitations merits attention. The data of our cross-sectional study were collected from a randomized controlled trial on the effectiveness of a cognitive-behavioral group training and not from an epidemiological study. As patients had to be referred to this trial by a healthcare provider and patients had to agree to take part in this treatment trial, our sample might have been a selective group. Selection during the referral could have both increased and decreased QoL and costs; possible selection biases could have been only referring patients with more severe symptoms and a high healthcare utilization for reasons such as being those most recognized by physicians as having UPS, or only referring patients with mild symptoms for reasons such as being best treatable in a relative short group training. Also, selection during the acquirement of patients’ informed consent might have both increased and decreased QoL and costs; possible selection biases could have been only getting informed consent from patients with more severe symptoms for reasons such as being the most burdensome and eager to try treatment, or, alternatively, only getting informed consent from patients with mild symptoms for reason such as being the most vital to show up at their first appointment. When looking at the characteristics of our patient group, our patient group seems to be rather a general than a selective sample. The characteristics of our sample can be described as mainly female, average age of 45 years, of whom 41% had a comorbid DSM-IV Axis I disorder and 29% had a comorbid DSM-IV Axis II disorder. These characteristics are in line with those found in other studies showing that UPS is more prevalent in women in their forties [4,5,7,9,48], and that 26 to 58% of the patients with UPS have a comorbid DSM-IV axis I disorder and 37 to 88,6% have a comorbid personality disorder [49-58]. Therefore, we believe that our patient group is a representative group for adult patients with UPS as defined in our study.

UPS in our study was defined as physical symptoms that fulfilled the DSM-IV criteria for an undifferentiated somatoform disorder or for a chronic pain disorder. This definition is stricter than the general used definition of UPS which is physical symptoms that cannot be explained on the basis of a known medical condition. The prevalence of both undifferentiated somatoform disorder and chronic pain disorder in general practices totals 14.6% [7], while the prevalence of UPS in primary care ranges from 20 to 74% depending on the definition and methods used to classify UPS [1]. As costs in our study were calculated using a prevalence of only 14.6%, the real total costs associated with UPS might be higher, and the mean cost per patient might be lower.

The QoL of patients with UPS was not adjusted for sociodemographic characteristics such as age, gender, education and living situation. Also, the QoL of the patient groups used as reference was not adjusted for sociodemographic characteristics. Not adjusting for sociodemographic characteristics might have affected our findings, as, in general, patients who are older or have a low education report a poorer QoL in the physical domain and patients who are female or not living with a partner report a poorer QoL in the mental domain [59]. Adjusting for the effects of sociodemographic characteristics seems to be artificial, as some illnesses, such as UPS and breast cancer, have different prevalences in various sociodemographic groups. By eliminating the effects of sociodemographic characteristics, results will visualize the burden of the illness itself and not the burden of patients, whereas treatments are indicated on the latter condition.

QoL and costs were not adjusted for comorbid mental and somatic disorders. As comorbidity reduces QoL [7,59] and increases costs [10], the QoL might have been lower and costs might have been higher due to the comorbidity prevalent in our study. However, isolating these effects would also be artificial, as patients with UPS suffer from many comorbid mental [54,60], and somatic disorders [8,10].

Another potential limitation is that QoL and costs were measured using self-report questionnaires. Potentially, self-report is subject to errors caused by recall difficulties. Recall is easier: 1) if the time-period between the event and the recall is shorter, a two-week interval having been suggested as best; and 2) if events fluctuate dramatically [61]. As the recall period of the questionnaire used on QoL and on the healthcare-related costs was four weeks, and as work-related events are likely to have fluctuated only slightly or moderately, errors in the recall may have followed.

The self-report data on the volume of healthcare consumption and production loss were extrapolated to estimate their volume at a one-year interval, an approach that would be appropriate only if the short intervals were chosen at random and were thereby generalizable. If this assumption was not met, the sample would have produced an inaccurate estimation of the volume of healthcare consumption and production loss.

Costs were estimated by multiplying the volume of healthcare consumption with reference prices and by multiplying the volume of production loss with the average productivity costs. Reference prices and productivity costs are average national cost prices and not actually paid prices. The advantage of the use of general cost prices is that they are a valuable contribution to decision-making at national level. However, the drawback is that they do not result in the actually paid costs and are less valuable for regional decision-making.

Work-related costs were based on a small sample of patients that resulted partly from our use of the friction-cost method, which reduced the number of employed patients in the cost estimations. The small sample of employed patients might also have been caused partly by the long duration of UPS in our study group. As our patients had endured their symptoms for a long time, their risk of losing paid employment due to symptoms had been increased.

The standard deviations around the cost estimates were large. This might be due to our relatively small sample size. However, despite a large population-based sample, Smit et al. [47] also found broad confidence intervals around the cost estimates in their study of the cost of mental disorders. Because cost-data have large standard errors, they concluded that this is a common finding in health-economic studies.

For the cost estimations, both conservative and less conservative methods were used. A less conservative method used in the cost estimation was to carry out the recommendation of the TiC-P manual to assess costs attributable to health problems in general, instead of those only attributable to the target disease to overcome difficulties for patients to distinguish between them. This might have led to an overestimation of costs. On the other hand, costs might also have been underrated by other more conservative methods used. A conservative method was the use of the friction-cost method, which led us to count only the cost of an absence from work of less than 23 weeks [24]. If we had counted absence from work irrespective of its duration (the so-called human-capital method), the number of patients with absenteeism would have doubled and the estimation of costs would have been higher. Also, the work-related cost due to presenteeism was estimated with a conservative method. The TiC-P manual provides two methods to calculate production loss due to presenteeism; the HLQ method and the Osterhaus method. In the HLQ method, the number of hours needed to catch up with work as reported by patients is used. In the Osterhaus method, the number of days of hindrance due to health problems and efficiency on these days as reported by patients is used. The study of Osterhaus et al. [62] showed that the HLQ method was more conservative and led to a lower cost estimation for presenteeism than the Osterhaus method. Finally, the work-related cost caused by substitution of domestic tasks had been estimated in a conservative manner as only the hours substituted by paid professionals were used in the estimation of costs. If the volume of total lost household productivity was used, then the number of patients who used substitution of domestic tasks would have been over fourfold and the estimated costs would have been higher.

Another strength of our study is that we examined QoL and costs in patients with UPS who were referred both by primary and secondary services. The resulting heterogeneous population made our results more generalizable than those of most studies that explored only the burden of UPS in primary care [4-10].

QoL and costs were assessed using generic measures. By using a generic measure for QoL which has the additional advantage of representing the different domains of QoL in one weighted score ‘utility’ , our study made it possible to compare patients with UPS with other patient groups to evaluate ‘who is in greatest need’. By using a generic measure for costs which assesses not only a broad spectrum of healthcare consumption but also production loss in paid and unpaid work, the study made it possible to compare patients with UPS with other patient groups to evaluate ‘who consumed which societal resources the most’.

The broad spectrum of cost-related outcomes, such as the number of healthcare visits, disability days, working hours lost to presenteeism in contrast to costs solely, have all the additional advantage to be internationally comparable, i.e. across countries which have different healthcare systems, healthcare services and labor markets. Presenting information on QoL and costs for different patient groups in quantified, unequivocal and comparable way helps politics and policymakers in their decision-making about allocation of healthcare resources.

## Conclusions

Information on QoL of specific patient groups and on the costs associated with them, and on how these relate to corresponding data in other patient groups is helpful in allocating healthcare resources. Our finding with regard to patients with UPS – that their QoL was one of the poorest of all patient groups – is a clear reminder that they are in great need and that the allocation of sufficient resources is justified. Interestingly, as their healthcare-related costs were among the highest, they already use a high level of resources. This seems to suggest that the solution is not increasing healthcare expenditure on patients with UPS. Instead, our findings raise the question whether current resources are used properly, and whether these resources should be reorganized to provide more effective and cost-effective treatments for patients with UPS.

When looking at our data of QoL reported by patients with UPS and the resources used by them, some hints about how to properly allocate resources might be deduced. Our data showed that patients had a more physical and social burden than an emotional one. Consistent with their burden, they searched for help in medical settings which seemed to provide expensive and not effective help. To reach patients with UPS, resources should be located in medical settings but the services provided in these settings should be improved.

An implementation of cognitive-behavioral therapy in medical settings seems to be rational, because cognitive-behavioral therapy is the most effective treatment [48,63,64]. As research has shown that the effectiveness of cognitive-behavioral therapy was difficult to replicate when conducted by general practitioners [65-70], the challenge of reorganizing resources might be to introduce psychologists specialized in cognitive-behavioral therapy in medical settings. This might stop the organization of healthcare services on the basis of the mind-body dualism, which seems to be old-fashioned and expensive way of organizing and allocating resources.

## Abbreviations

MID: Minimally important difference; PPPY: Per patient per year; QoL: Quality of life; SCID-I: Structured Clinical Interview for DSM-IV Axis I Disorders; SF-36: 36-item medical outcomes study Short-Form general health survey; SF-6D: A scorings algorithm for the SF-36 to calculate a ‘utility’; TiC-P: Trimbos/iMTA Questionnaire for Costs associated with Psychiatric Illness; UPS: Unexplained physical symptoms.

## Competing interests

The authors declare that they have no competing interests.

## Authors’ contributions

LNLZ developed the original idea of the study, implemented the study, conducted the analyses and drafted the manuscript. All authors read and corrected draft versions. All authors approved the final version of this manuscript.

## Authors’ information

LNLZ is Ph.D., clinical psychologist, psychotherapist and cognitive behavioral therapist. MAGS is professor and principal investigator of the Department of Medical psychology, Academic Medical Center in Amsterdam, the Netherlands. CGK is Ph.D., psychiatrist and psychoanalytist. AvtS is Ph.D., assistant professor and psychotherapist. JJVB is professor and head of the section Medical Psychology and Psychotherapy of the department of Psychiatry of Erasmus Medical Center in Rotterdam, and senior investigator at the Viersprong Institute for studies on Personality Disorders (VISPD) of the ‘Viersprong’ in Halsteren, the Netherlands.

## Pre-publication history

The pre-publication history for this paper can be accessed here:

http://www.biomedcentral.com/1472-6963/13/520/prepub

## Supplementary Material

Additional file 1 **Quality of life in different reference populations.** Comparison of the SF-36 subscale means of patients with UPS with those found in patients with major depression, in patients with cancer, and in the general population. Click here for file

Additional file 2 **Utilities in different reference populations.** Comparison of the SF-6D median of patients with UPS with those found in patients with mental disorders and chronic physical conditions and in the general population. Click here for file

Additional file 3 **Total number of patients with UPS, their annual healthcare expenditures and the percentage of total Dutch annual healthcare expenditure associated with UPS.** Calculation of the percentage of total Dutch annual healthcare expenditure associated with UPS Click here for file

Additional file 4 **Distribution of total Dutch healthcare expenditures over all main classifications.** Comparison of the percentage of total Dutch annual healthcare expenditure associated with UPS with those found in all main disease categories. Click here for file

Additional file 5 **Healthcare expenditures over different diseases.** Comparison of the percentage of total Dutch annual healthcare expenditure associated with UPS with those found in specific diseases and the general population. Click here for file

Additional file 6 **Disability days per person per year and percentage of absenteeism in different reference populations.** Comparison of the work-related cost due to absenteeism of patients with UPS with those found in the general population, the healthy workforce, and the workforce with chronic illness, using the mean number of disability days and the mean percentage of absenteeism per patient per year. Click here for file

Additional file 7 **Cost of presenteeism per person per year in different reference populations.** Comparison of the work-related cost due to presenteeism of patients with UPS with that found in the general population and the workforce with chronic illness. Click here for file
