# Supplementary figures and images for: Patients with unexplained physical symptoms have poorer quality of life and higher costs than other patient groups: a cross-sectional study on burden
Source: BMC Health Serv Res. 2013 Dec 17;13:520. doi: 10.1186/1472-6963-13-520 (PMC3878564; doi:10.1186/1472-6963-13-520)

## Additional file 1 Quality of life in different reference populations


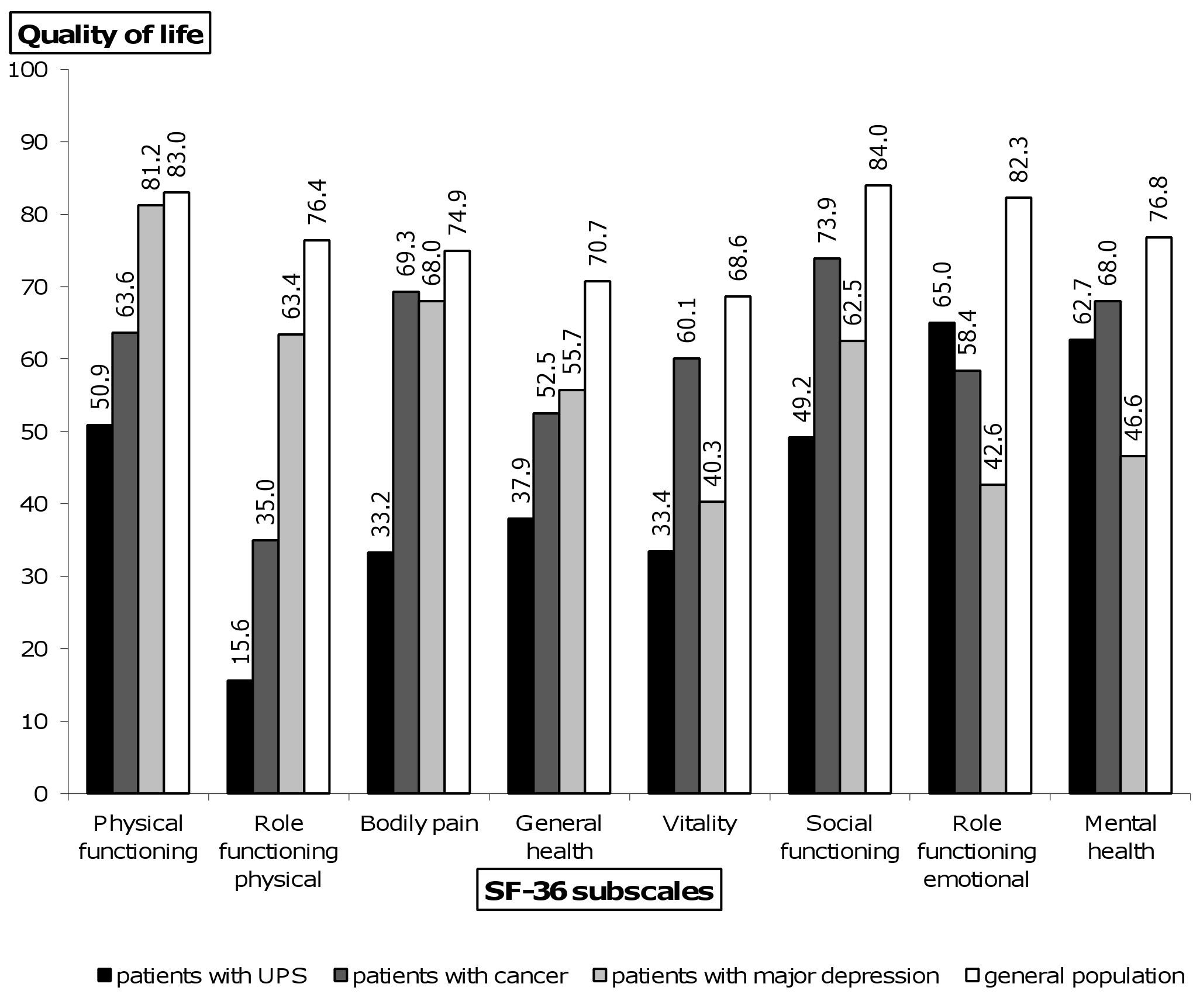

Supplement: Additional file 1 — Quality of life in different reference populations. Comparison of the SF-36 subscale means of patients with UPS with those found in patients with major depression, in patients with cancer, and in the general population. [file 1472-6963-13-520-S1.docx]
