# Supplementary material for: Patients with unexplained physical symptoms have poorer quality of life and higher costs than other patient groups: a cross-sectional study on burden
Source: BMC Health Serv Res. 2013 Dec 17;13:520. doi: 10.1186/1472-6963-13-520 (PMC3878564; doi:10.1186/1472-6963-13-520)
Supplement: Additional file 2 — Utilities in different reference populations. Comparison of the SF-6D median of patients with UPS with those found in patients with mental disorders and chronic physical conditions and in the general population. [file 1472-6963-13-520-S2.docx]

**Additional file 2 Utilities in different reference populations**

| **Reference group** | **N** | **Utility (median)** |
| --- | --- | --- |
| ***General population [***[***1***](#_ENREF_1)***]*** | **1,005** | **0.782** |
| ***Mental disorder*** | | |
| alcohol dependence or misuse [[2](#_ENREF_2)] | 81 | 0.737 |
| any substance misuse [[2](#_ENREF_2)] | 122 | 0.723 |
| specific phobia [[2](#_ENREF_2)] | 232 | 0.698 |
| generalized anxiety disorder [[2](#_ENREF_2)] | 129 | 0.681 |
| any anxiety disorder [[2](#_ENREF_2)] | 659 | 0.660 |
| panic disorder [[2](#_ENREF_2)] | 253 | 0.606 |
| dysthymia [[2](#_ENREF_2)] | 115 | 0.603 |
| social phobia [[2](#_ENREF_2)] | 64 | 0.599 |
| *Unexplained Physical Symptoms: UPS* | *157* | *0.568* |
| any mood disorder [[2](#_ENREF_2)] | 476 | 0.547 |
| major depressive disorder [[2](#_ENREF_2)] | 332 | 0.527 |
| ***Chronic physical condition*** | | |
| heart attack [[2](#_ENREF_2)] | 171 | 0.755 |
| cancer (breast, lung, colorectal) [[3](#_ENREF_3)] | 184 | 0.74 |
| diabetes [[2](#_ENREF_2)] | 373 | 0.738 |
| high blood pressure [[2](#_ENREF_2)] | 1,075 | 0.737 |
| cardiovascular diseases [[2](#_ENREF_2)] | 496 | 0.724 |
| chronic pain [[2](#_ENREF_2)] | 2,496 | 0.723 |
| heart diseases [[2](#_ENREF_2)] | 457 | 0.723 |
| respiratory conditions [[2](#_ENREF_2)] | 467 | 0.723 |
| chronic bronchitis [[2](#_ENREF_2)] | 347 | 0.705 |
| asthma [[2](#_ENREF_2)] | 227 | 0.705 |
| arthritis [[2](#_ENREF_2)] | 1,480 | 0.681 |
| neck pain [[2](#_ENREF_2)] | 1,471 | 0.675 |
| back pain [[2](#_ENREF_2)] | 1,484 | 0.669 |
| migraines [[2](#_ENREF_2)] | 717 | 0.657 |
| *Unexplained Physical Symptoms: UPS* | *157* | *0.568* |

## References

1. Kontodimopoulos N, Pappa E, Papadopoulos AA, Tountas Y, Niakas D: **Comparing SF-6D and EQ-5D utilities across groups differing in health status**. *Qual Life Res* 2009, **18**(1):87-97.

2. Fernández A, Saameño JÁB, Pinto-Meza A, Luciano JV, Autonell J, Palao D, Salvador-Carulla L, Campayo JG, Haro JM, Serrano A: **Burden of chronic physical conditions and mental disorders in primary care**. *Br J Psychiatry* 2010, **196**(4):302-309.

3. Teckle P, Peacock S, McTaggart-Cowan H, Van der Hoek K, Chia S, Melosky B, Gelmon K: **The ability of cancer-specific and generic preference-based instruments to discriminate across clinical and self-reported measures of cancer severities**. *Health and Quality of Life Outcomes* 2011, **9**:106.
