# Supplementary material for: Patients with unexplained physical symptoms have poorer quality of life and higher costs than other patient groups: a cross-sectional study on burden
Source: BMC Health Serv Res. 2013 Dec 17;13:520. doi: 10.1186/1472-6963-13-520 (PMC3878564; doi:10.1186/1472-6963-13-520)
Supplement: Additional file 3 — Total number of patients with UPS, their annual healthcare expenditures and the percentage of total Dutch annual healthcare expenditure associated with UPS. Calculation of the percentage of total Dutch annual healthcare expenditure associated with UPS [file 1472-6963-13-520-S3.docx]

**Additional file 3 Calculation of the percentage of total Dutch annual healthcare expenditure associated with UPS**

| Sub-group | **Percentage or number** | **Estimator** |
| --- | --- | --- |
| *Prevalence of undifferentiated somatoform disorder in general practices [*[*1*](#_ENREF_1)*]* | *13.0%* |  |
| *Prevalence of chronic pain disorder in general practices [*[*1*](#_ENREF_1)*]* | *1.6%* |  |
| Prevalence of Unexplained Physical Symptoms (UPS) in general practices |  | 14.6% |
| Percentage of the general population visiting a general practice in 2007 [[2](#_ENREF_2)] | *72.4%* | 72.4% |
| *Number of people in the general population aged between 20 and 40 years in 2007 [*[*3*](#_ENREF_3)*]* | *4,319,136* |  |
| *Number of people in the general population aged between 40 and 65 years in 2007 [*[*3*](#_ENREF_3)*]* | *5,713,401* |  |
| Total number of people in the general population aged between 20 and 65 years |  | 10,032,537 |
| Total number of patients with Unexplained Physical Symptoms (UPS) |  | **1,060,479** |
| Mean medical costs per patient with Unexplained Physical Symptoms (UPS) per year |  | €3,122.93 |
| Total annual healthcare expenditures related to Unexplained Physical Symptoms (UPS) |  | **€3,311,800,000** |
| Total Dutch annual healthcare expenditures [[4](#_ENREF_4)] | €74,447,000,000 |  |
| The percentage of total annual healthcare expenditures related to UPS |  | **4.4%** |

## References

1. De Waal MWM, Arnold IA, Eekhof JAH, Van Hemert AM: **Somatoform disorders in general practice: prevalence, functional impairment and comorbidity with anxiety and depressive disorders**. *Br J Psychiatry* 2004, **184**(6):470-476.

2. Statistics N: **Verschillen in cijfers over huisartscontacten tussen POLS-Gezondheid en de statistiek 'Geregistreerde contacten met de huisarts'**. In*.* Den Haag/Heerlen: Statistics Netherlands/Centraal Bureau voor de Statistiek; 2009.

3. **Bevolking; kerncijfers (2007)** [<http://statline.cbs.nl/StatWeb/publication/?DM=SLNL&PA=37296ned&D1=0-51&D2=57&VW=T>]

4. Slobbe LCJ, Smit JM, Groen J, Poos MJJC, Kommer GJ: **Kosten van ziekten in Nederland 2007: trends in de Nederlandse zorguitgaven 1999-2010**. In: *Zorg voor euro's.* Bilthoven: Rijksinstituut voor Volksgezondheid en Milieu (RIVM)/Centraal Bureau voor de Statistiek (CBS); 2011: RIVM-rapportnummer 270751023/270752011.
