# Supplementary material for: Patients with unexplained physical symptoms have poorer quality of life and higher costs than other patient groups: a cross-sectional study on burden
Source: BMC Health Serv Res. 2013 Dec 17;13:520. doi: 10.1186/1472-6963-13-520 (PMC3878564; doi:10.1186/1472-6963-13-520)
Supplement: Additional file 4 — Distribution of total Dutch healthcare expenditures over all main classifications. Comparison of the percentage of total Dutch annual healthcare expenditure associated with UPS with those found in all main disease categories. [file 1472-6963-13-520-S4.docx]

**Additional file 4 Distribution of total Dutch healthcare expenditures over all main classifications [**[**1**](#_ENREF_1)**]**

| **Classification in this study** | **Costs in million €** | **% of the total expenditures** |
| --- | --- | --- |
| *Unexplained Physical Symptoms: UPS* | *3,312* | *4.4%* |
| **Main classification** | **Costs in million €** | **% of the total expenditures** |
| Blood and blood-forming organs | 236 | 0.3% |
| Congenital malformation | 250 | 0.3% |
| Perinatal diseases | 419 | 0.6% |
| Skin and subcutaneous | 802 | 1.1% |
| Inflammation diseases and parasitic diseases | 1,064 | 1.4% |
| Pregnancy, childbirth and childbed | 1,555 | 2.1% |
| Endocrine, dietary and metabolic diseases | 1,707 | 2.3% |
| Urogenital system | 1,907 | 2.6% |
| Accident, injuries and poisoning | 2,141 | 2.9% |
| Respiratory system | 2,618 | 3.5% |
| Blastomas (cancer and benign tumors) | 3,423 | 4.6% |
| Nervous system and sense organs | 3,981 | 5.3% |
| Symptoms and incompletely described syndromes | 4,093 | 5.5% |
| Digestion system | 4,879 | 6.6% |
| Musculoskeletal system and connective tissue | 4,950 | 6.6% |
| Cardiovascular system | 6,911 | 9.3% |
| Psychiatric disorders | 15,895 | 21.4% |
| Not allocated/not illness related | 17,615 | 23.7% |
| **Total Dutch health care expenditure** | **74,447** | **100.0%** |

## References

1. Slobbe LCJ, Smit JM, Groen J, Poos MJJC, Kommer GJ: **Kosten van ziekten in Nederland 2007: trends in de Nederlandse zorguitgaven 1999-2010**. In: *Zorg voor euro's.* Bilthoven: Rijksinstituut voor Volksgezondheid en Milieu (RIVM)/Centraal Bureau voor de Statistiek (CBS); 2011: RIVM-rapportnummer 270751023/270752011.
