# Supplementary material for: Patients with unexplained physical symptoms have poorer quality of life and higher costs than other patient groups: a cross-sectional study on burden
Source: BMC Health Serv Res. 2013 Dec 17;13:520. doi: 10.1186/1472-6963-13-520 (PMC3878564; doi:10.1186/1472-6963-13-520)
Supplement: Additional file 5 — Healthcare expenditures over different diseases. Comparison of the percentage of total Dutch annual healthcare expenditure associated with UPS with those found in specific diseases and the general population. [file 1472-6963-13-520-S5.docx]

**Additional file 5 Healthcare expenditures over different diseases [**[**1**](#_ENREF_1)**]**

| **Reference group** | **% of the total expenditures** |
| --- | --- |
| ***General population*** | |
| general population aged between 15 and 65 years | 48.9% |
| men aged between 15 and 65 years | 23.3% |
| women aged between 15 and 65 years | 25.6% |
| ***Psychiatric disorder (all disorders included)*** | |
| psychotic disorder (excluding schizophrenia) | 0.1% |
| personality disorder | 0.5% |
| anxiety disorder | 0.6% |
| schizophrenia | 1.2% |
| mood disorder | 1.3% |
| alcohol and drugs dependence/misuse | 1.4% |
| other psychiatric disorders | 4.0% |
| *(Unexplained Physical Symptoms: UPS* | *4.4%)* |
| dementia | 4.7% |
| mental retardation (including Down syndrome) | 7.6% |
| ***Chronic physical condition (diseases with costs in the bottom and top five diseases included)*** | |
| *–bottom five–* | |
| congenital anomalies of nervous system | 0.02% |
| meningitis | 0.04% |
| hepatitis | 0.04% |
| malignant neoplasm of ovary and other uterine adnexa | 0.05% |
| chronic liver disease and cirrhosis | 0.05% |
| *–in between–* | |
| Parkinson's disease | 0.3% |
| multiple sclerosis | 0.3% |
| cancer (colon, rectum, rectosigmoid junction, or anus) | 0.4% |
| rheumatoid arthritis and other inflammatory polyarthropathies | 0.7% |
| osteoarthritis and allied disorders | 1.0% |
| disorders of soft tissues | 1.1% |
| hypertension | 1.2% |
| bronchitis, emphysema, asthma, bronchiectasis, extrinsic allergic alveolitis, chronic airway obstruction (including COPD) | 1.3% |
| diabetes mellitus (including diabetic complications) | 1.4% |
| *–top five–* | |
| rheumatic heart disease, diseases of endocardial structures, pulmonary circulation pericardium and endocardium, cardiomyopathy, conduction disorders, cardiac dysrhythmias | 1.8% |
| diffuse diseases of musculoskeletal system and connective tissue | 2.0% |
| cerebrovascular diseases (stroke) | 2.2% |
| diseases of hard tissues of teeth | 2.4% |
| chronic ischemic heart diseases | 2.4% |
| *(Unexplained Physical Symptoms: UPS* | *4.4%)* |

## References

1. Slobbe LCJ, Smit JM, Groen J, Poos MJJC, Kommer GJ: **Kosten van ziekten in Nederland 2007: trends in de Nederlandse zorguitgaven 1999-2010**. In: *Zorg voor euro's.* Bilthoven: Rijksinstituut voor Volksgezondheid en Milieu (RIVM)/Centraal Bureau voor de Statistiek (CBS); 2011: RIVM-rapportnummer 270751023/270752011.
