# Supplementary material for: Patients with unexplained physical symptoms have poorer quality of life and higher costs than other patient groups: a cross-sectional study on burden
Source: BMC Health Serv Res. 2013 Dec 17;13:520. doi: 10.1186/1472-6963-13-520 (PMC3878564; doi:10.1186/1472-6963-13-520)
Supplement: Additional file 6 — Disability days per person per year and percentage of absenteeism in different reference populations. Comparison of the work-related cost due to absenteeism of patients with UPS with those found in the general population, the healthy workforce, and the workforce with chronic illness, using the mean number of disability days and the mean percentage of absenteeism per patient per year. [file 1472-6963-13-520-S6.docx]

**Additional file 6 Disability days per person per year and percentage of absenteeism in different reference populations**

| **Study group** | **N** | **Mean number disability days per year** | **Percentage of absenteeism** |
| --- | --- | --- | --- |
| *Unexplained Physical Symptoms: UPS* | | | |
| workforce | *73* | *67.3* | *39.3* |
| workforce within friction period | *58* | *42.6* | *23.5* |
| **Reference group** | | | |
| *General population* | | | |
| workforce [[1](#_ENREF_1)] | 23,000 | 7.5 | 4.2 |
| working men [[1](#_ENREF_1)] | 12,489 | 7.4 | 3.7 |
| working women [[1](#_ENREF_1)] | 10,511 | 7.7 | 4.7 |
| *Healthy workforce* | | | |
| men and women [[2](#_ENREF_2)] | 14,697 | *not available* | 2.8 |
| men [[2](#_ENREF_2)] | 7,980 | *not available* | 2.4 |
| women [[2](#_ENREF_2)] | 6,717 | *not available* | 3.3 |
| *Workforce with chronic illnesses* | | | |
| men and women [[2](#_ENREF_2)] | 8,303 | *not available* | 7.4 |
| men [[2](#_ENREF_2)] | 4,509 | *not available* | 6.8 |
| women [[2](#_ENREF_2)] | 3,794 | *not available* | 8.0 |
| *Workforce with specific chronic illness* | | | |
| bipolar disorder [[3](#_ENREF_3)] | 30 | 55.5 | *not available* |
| personality disorder [[4](#_ENREF_4)] | 743 | 27.7 | *not available* |

## References

1. Klein Hesselink J, Hooftman W, Koppes L: **Ziekteverzuim in Nederland in 2010**. In*.* Hoofddorp: TNO; 2012.

2. Jehoel-Gijsbers Gr: **Beperkt aan het werk: rapportage ziekteverzuim, arbeidsongeschiktheid en arbeidsparticipatie**. In*.* Den Haag: Sociaal en Cultureel Planbureau; 2010.

3. Hakkaart-van Roijen L, Hoeijenbos MB, Regeer EJ, Ten Have M, Nolen WA, Veraart CPWM, Rutten FFH: **The societal costs and quality of life of patients suffering from bipolar disorder in the Netherlands**. *Acta Psychiatr Scand* 2004, **110**(5):383-392.

4. Soeteman DI, Hakkaart-van Roijen L, Verheul R, Busschbach JJV: **The economic burden of personality disorders in mental health care**. *J Clin Psychiatry* 2008, **69**(2):259-265.
