# Supplementary material for: Patients with unexplained physical symptoms have poorer quality of life and higher costs than other patient groups: a cross-sectional study on burden
Source: BMC Health Serv Res. 2013 Dec 17;13:520. doi: 10.1186/1472-6963-13-520 (PMC3878564; doi:10.1186/1472-6963-13-520)
Supplement: Additional file 7 — Cost of presenteeism per person per year in different reference populations. Comparison of the work-related cost due to presenteeism of patients with UPS with that found in the general population and the workforce with chronic illness. [file 1472-6963-13-520-S7.docx]

**Additional file 7 Cost of presenteeism per person per year in different reference populations**

|  | **N** | **Mean annual cost per person with paid work due to presenteeism^*†^** | |
| --- | --- | --- | --- |
| **Study group** |  | € | US$ |
| *Unexplained Physical Symptoms: UPS* | | | |
| workforce | *73* | 1,899 | *2,520* |
| **Reference workforce group** | | | |
| *General population* | | | |
| healthy study controls [[1](#_ENREF_1)] | 61 | 572 | *759* |
| *Psychiatric disorder* | | | |
| bipolar disorder [[2](#_ENREF_2)] | 30 | *298* | 395 |
| personality disorder [[3](#_ENREF_3)] | 743 | 960 | *1,274* |
| *Chronic physical disease* | | | |
| rheumatoid arthritis [[1](#_ENREF_1)] | 62 | 4,108 | *5,450* |

**^*^**The exchange rate €/$ on 25 March 2012 was 1.3268.

**^†^**Costs converted to another currency are written in *italics.*

## References

1. Braakman-Jansen LMA, Taal E, Kuper IH, Van de Laar MAFJ: **Productivity loss due to absenteeism and presenteeism by different instruments in patients with RA and subjects without RA**. *Rheumatology (Oxford)* 2012, **51**(2):354-361.

2. Hakkaart-van Roijen L, Hoeijenbos MB, Regeer EJ, Ten Have M, Nolen WA, Veraart CPWM, Rutten FFH: **The societal costs and quality of life of patients suffering from bipolar disorder in the Netherlands**. *Acta Psychiatr Scand* 2004, **110**(5):383-392.

3. Soeteman DI, Hakkaart-van Roijen L, Verheul R, Busschbach JJV: **The economic burden of personality disorders in mental health care**. *J Clin Psychiatry* 2008, **69**(2):259-265.
